# Safety of *Pelargonium* extract EPs 7630 in young children with acute bronchitis

**DOI:** 10.3389/fped.2023.1107984

**Published:** 2023-02-14

**Authors:** Wolfgang Kamin, Ulrich Behre, Klaus Helm, Birgit Reling, Petra Funk, Fathi Abdul Malek

**Affiliations:** ^1^ Children’s Hospital, Evangelic Hospital, Hamm, Germany; ^2^ Faculty of Medicine, Pomeranian Medical University, Szczecin, Poland; ^3^ Pediatric Practice, Kehl, Germany; ^4^ Pediatric Practice, Detmold, Germany; ^5^ General Practice, Remscheid, Germany; ^6^ Research and Development, Dr. Willmar Schwabe GmbH & Co. KG, Karlsruhe, Germany

**Keywords:** acute bronchitis, children, Pelargonium sidoides DC. root extract (EPs® 7630), clinical trial, safety

## Abstract

**Background:**

The efficacy and tolerability of *Pelargonium sidoides* DC. root extract EPs 7630 in children with acute bronchitis (AB) have been widely demonstrated. We investigated the safety and tolerability of a syrup formulation and an oral solution in pre-school children.

**Methods:**

In an open-label, randomized clinical trial (EudraCT number 2011-002652-14), children aged 1–5 years suffering from AB received EPs 7630 syrup or solution for 7 days. Safety was assessed by frequency, severity, and nature of adverse events (AE), vital signs, and laboratory values. Outcome measures for evaluating the health status were the intensity of coughing, pulmonary rales, and dyspnea, measured by the short version of the Bronchitis Severity Scale (BSS-ped), further symptoms of the respiratory infection, general health status according to the Integrative Medicine Outcomes Scale (IMOS), and satisfaction with treatment according to the Integrative Medicine Patient Satisfaction Scale (IMPSS).

**Results:**

591 children were randomized and treated with syrup (*n* = 403) or solution (*n* = 188) for 7 days. In both treatment groups, the number of adverse events was similarly low and revealed no safety concerns. The most frequently observed events were infections (syrup: 7.2%; solution: 7.4%) or gastrointestinal disorders (syrup: 2.7%; solution: 3.2%). After one week's treatment, more than 90% of the children experienced an improvement or remission of the symptoms of the BSS-ped. Further respiratory symptoms decreased similarly in both groups. At Day 7, more than 80% of the whole study population had completely recovered or showed a major improvement as assessed by the investigator and the proxy, respectively. Parents were “very satisfied” or “satisfied” with the treatment in 86.1% of patients in the combined syrup and solution group.

**Conclusion:**

Both pharmaceutical forms, EPs 7630 syrup and oral solution, were shown to be equally safe and well tolerated in pre-school children suffering from AB. Improvement of health status and of complaints were similar in both groups.

## Introduction

Acute bronchitis (AB) is one of the most common infections reported in children under 5 years of age and a leading cause of hospitalization (1). It is predominantly attributable to a viral infection (2); the most commonly identified viruses are rhinovirus, enterovirus, influenza A and B, parainfluenza virus, coronavirus, human metapneumovirus, and respiratory syncytial virus (2, 3).

A treatment option for acute respiratory tract infections (ARTIs) authorized in many countries worldwide is EPs 7630,^1^ a herbal extract from the roots of *Pelargonium sidoides* DC. In-vitro-experiments with EPs 7630 and isolated constituents have demonstrated pharmacological activities including moderate direct antiviral and antibacterial action and notable immune-modulatory capabilities. The latter involve activation of the MAP kinase pathway (4) and subsequent regulation of different cytokines such as tumor necrosis factor α, interferon-β, or interleukin-22, depending on the experimental context (5, 6). In an animal study, antitussive, secretolytic, and anti-inflammatory effects of EPs 7630 were detected following the oral administration at human equivalent doses (7). Moreover, EPs 7630 interferes with the replication of respiratory viruses (8) and reduces rhinovirus infection to human bronchial cells (9, 10). In several *in vitro* experiments, EPs 7630 was also shown to affect SARS-CoV-2 replication and innate immune responses in the human lung cell line Calu-3 (11, 12).

Over the last 25 years, EPs 7630 has been investigated for the treatment of ARTIs in more than 30 clinical trials and the efficacy and tolerability of EPs 7630 in patients with various ARTIs have been shown (13–19). Most recently, a randomized, single-blind, placebo-controlled study with 164 patients aged 1 to 18 years showed that *Pelargonium sidoides* extract is effective in relieving the symptom burden of the disease in uncomplicated upper respiratory tract infections (20). Moreover, eight randomized, double-blind, placebo-controlled trials involving 1,253 children and adolescents demonstrated the safety and efficacy of EPs 7630 for the treatment of ARTIs (21). A meta-analysis conducted with five randomized controlled trials comprising a total of 990 patients revealed further evidence for efficacy and safety in the treatment of respiratory tract infections in children and adolescents (22). A dedicated literature review focusing on pre-school children identified seven studies with 1,097 children up to 5 years old who were exposed to EPs 7630 (23). The authors concluded that EPs 7630 is efficacious in acute bronchitis and the effectiveness in other ARTIs is supported. No safety concerns were identified.

Based on clinical evidence for efficacy and safety of EPs 7630 in children, marketing authorizations for this age group were granted by authorities in Europe and worldwide. The EPs 7630 syrup formulation has been introduced in several countries for children from the age of 1 year suffering from AB or other ARTIs. The present post-authorization safety study was performed to meet an obligation of the drug regulatory authority in Germany (Bundesinstitut für Arzneimittel und Medizinprodukte). The objective was to assess the tolerability and safety of EPs 7630 syrup in 1- to 5-year old children suffering from acute bronchitis (AB) and to compare the results with those for EPs 7630 solution. No difference was expected regarding patient safety since the active substance and the excipients of both dosage forms possess well-established tolerability. Hepatic enzyme activities were of special interest in the context of hepatic involvement described in children and adolescents suffering from ARTIs (3).

## Materials and methods

### Study design, schedule, and ethical conduct

The study was performed as an open label, randomized, active controlled multicenter trial. At the screening visit, a general physical examination was carried out. Eligible participants were randomized to receive either EPs 7630 syrup or solution for 7 days. A study exit examination was performed at the end of the treatment period. AEs were documented during the trial and up to 7 days after the last intake of the study medication.

### Participants

Eligible patients were 1 to 5 years of age, with at least 2 of the AB symptoms coughing, pulmonary rales at auscultation, or dyspnea which started within the last 72 h before screening. Major exclusion criteria were antibiotic medication or treatment with anticoagulants within 6 weeks prior to inclusion, any indication for antibiotic treatment, diagnosis, or suspicion of airway diseases other than AB, otitis media, foreign body aspiration, bronchial asthma, recurrent bronchitis, allergic rhinitis, or other allergic diseases, known or suspected hypersensitivity to any constituent of the investigational products, bleeding tendency, gastro-esophageal reflux disease, or other known or suspected gastrointestinal disorder.

### Interventions and randomization

EPs 7630 is an extract from the roots of *Pelargonium sidoides*, drug-extract ratio 1 : 8–10, extraction solvent ethanol 11% (w/w). 10 g (=9.75 ml) of oral solution contain 8.0 g extract, 100 g (=93.99 ml) of syrup contain 0.25 g of the dried extract (obtained from the liquid extract by drying). 2.5 ml of syrup are equivalent to 10 drops of the solution. EPs 7630 solution and syrup both contain glycerol 85% as a further pharmaceutical excipient.

Study participants were randomized at a ratio of 2 : 1 to receive EPs 7630 syrup (2.5 ml, as measured by the provided measuring cup) or solution (10 drops) 3 times daily. The assessment of compliance was based on unused investigational product returned at the second visit (Day 7, termination). The reason for randomizing 2/3 of the patients to the syrup group was to maximize the number of participants who received the novel formulation while safety data for EPs 7630 solution had already been obtained in the population of interest during previous clinical trials (23).

Randomization was performed in permutated blocks with block sizes of 3. The random code list was generated by a member of the sponsor's biometrics department otherwise not involved in the project, using the validated SAS macro RANSCH (24). To minimize allocation bias, the information about the medication corresponding to the randomization numbers was included in sealed envelopes to be opened only after the patient's enrolment, and the random block size was withheld from the trial sites. Randomization numbers were allocated sequentially.

### Outcomes

Safety assessment was based on the frequency, severity, and nature of AEs, changes in vital signs (heart rate, respiration rate, body temperature) and changes in laboratory parameters (alanine aminotransferase, ALT; aspartate aminotransferase, AST; gamma-glutamyltranspeptidase, γGT; C-reactive protein, CRP). Laboratory values out of the normal ranges were assessed by the investigator if they were clinically relevant. At the final visit, participants and their legal guardians were questioned by the investigator for AEs in a general manner, i.e., without asking for certain events.

An AE which was reported spontaneously by the participants or legal guardians, or which was observed, had to be classified by the investigator according to the available data to one of four relationship categories (probable, possible, unlikely, no relationship), recorded, and monitored irrespective of causal relationship.

The patients’ condition was evaluated by means of the BSS-ped, which is a validated physician-rated assessment scale including the symptoms coughing, pulmonary rales at auscultation, and dyspnea (25, 26). The three symptoms were rated on a 5-point scale ranging from “not present” (0 points) to “very severe” (4 points). A total score was computed as the sum of the individual item scores. Using the same scale, nasal discharge, nasal congestion, sneezing, congestion of pharyngeal mucosa, hoarseness, loss of appetite, and sleep disorder were assessed as further symptoms related to respiratory infection. Specific guidance for the grading of each symptom was given in the study protocol. Hoarseness was rated by the physician at the visit, sleep disorder was rated for the preceding night; all other symptom ratings refer to the last 24 h preceding Day 7. The overall recovery from AB and the patients' (and caregivers') satisfaction with treatment outcome were assessed using the Integrative Medicine Outcomes Scale (IMOS) and the Integrative Medicine Patient Satisfaction Scale (IMPSS), respectively (27).

Treatment compliance was assessed by determining the remaining amount of trial medication at study exit.

### Sample size and statistical methods

It was intended to randomize 600 patients (syrup 400; solution 200). Since the study's primary objective was an analysis of safety and tolerability, no formal hypotheses were tested, and the data were analysed descriptively in line with the ICH E9 guideline (28). For a sample size of 400 patients treated with EPs 7630 syrup, the upper bound of the one-sided 97.5% confidence interval (CI) for the probability of observing an adverse event in the population of interest that has not been observed in the study population is 0.009, i.e., events that appear in at least 1 of 100 treated patients in the population had a probability of 97.5% for being observed at least once during the trial. Comparison of the treatment groups regarding the incidence, type, and severity of AEs was performed descriptively.

Laboratory measures and vital signs were analyzed for shifts in the mean and for deviations from the applicable reference ranges. Changes in BSS-ped and further symptoms related to respiratory infection as well as treatment outcome and satisfaction were analyzed using descriptive data analysis. For the items of the BSS-ped, remission was defined as a value of 0 (no symptoms) at treatment end following a value >0 at baseline. Improvement was defined as any score decrease other than remission. For the assessments of further symptoms related to respiratory infection, missing item scores at baseline were replaced by the medians of the non-missing values within each treatment group, and the baseline values were carried forward when post-treatment data were missing.

Safety and tolerability were analyzed based on the safety analysis set, which consisted of participants who had taken the investigational product at least once. BSS-ped was analyzed in the full analysis set, which included all patients with any post-baseline data. The per-protocol analysis set comprised participants who had completed the study without major protocol deviations.

## Results

### Study participants

**Figure 1:**
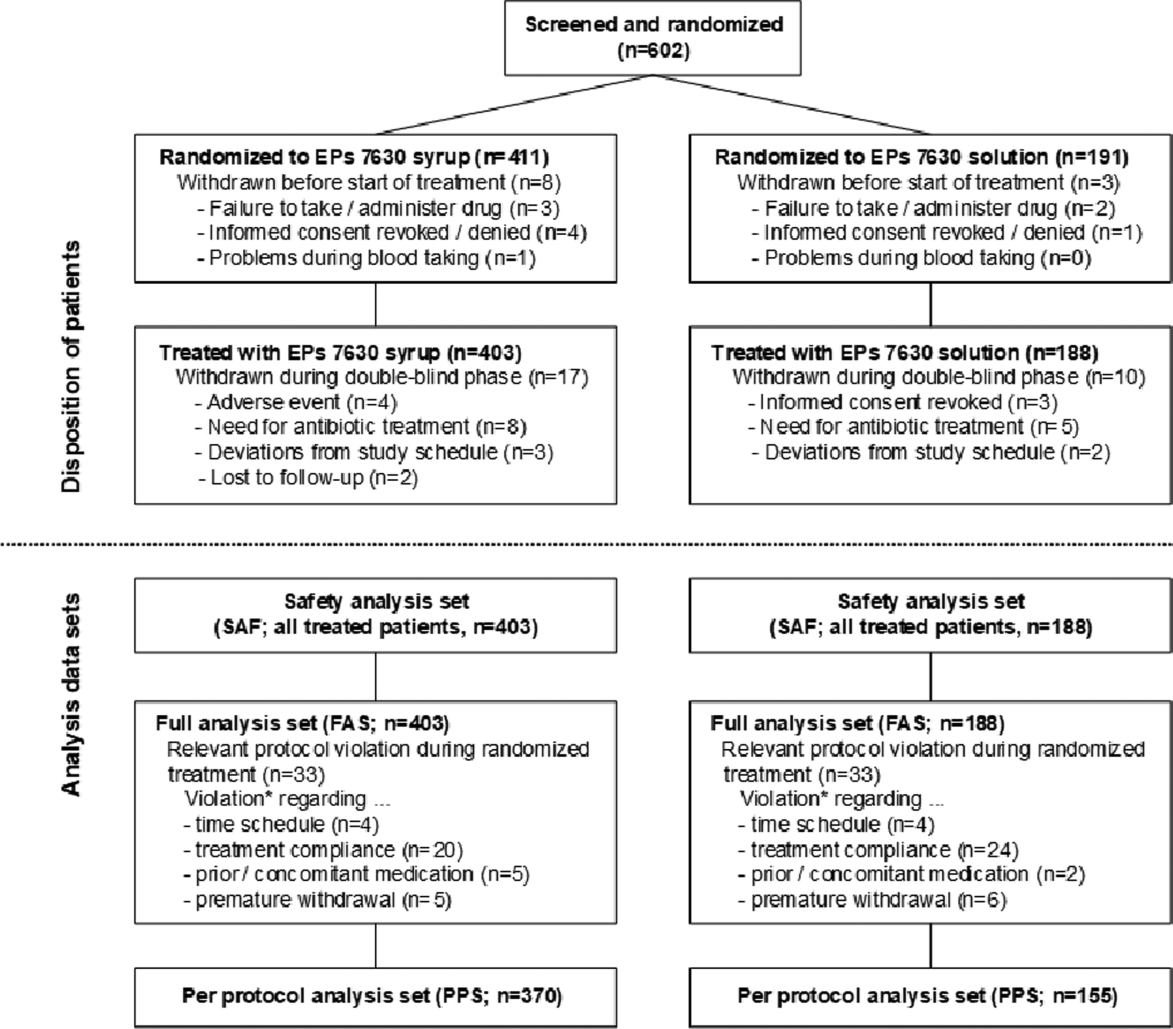
Disposition of patients, analysis data sets. *Multiple reasons possible.

602 male or female children were screened and randomized to treatment with EPs 7630 syrup (*n* = 411) or EPs 7630 solution (*n* = 191) in 35 general and pediatric practices (Figure 1). Eleven patients terminated trial participation before receiving any investigational treatment. The main reasons were failure to administer or to take any dose of the study drug, or refusal of the second parent to provide informed consent/withdrawal of consent before the first dose.

Overall, 591 patients were treated with syrup (*n* = 403) or solution (*n* = 188). 27 patients (syrup: 17 of 403, 4.2%; solution: 10 of 188, 5.3%) were withdrawn before the scheduled treatment end. 4 patients withdrew prematurely due to AEs, i.e., angina tonsillaris, otitis media, pneumonia, and bronchopneumonia, all of which were considered unrelated to the investigational products. Other reasons for premature withdrawal were antibiotic treatment (13 patients), relevant deviations from the specified visit days (5 patients), withdrawal of informed consent (3 patients), and loss to follow-up (2 patients). All treated patients participated in the final examination except for two who were lost to follow-up.

All treated patients were analyzed for safety and for changes in their bronchitis-related health status (Figure 1). Major protocol deviations leading to an exclusion from the per-protocol analysis set were an interval of less than 6 or more than 10 days between baseline and the end of study visit, premature withdrawal unrelated to efficacy or tolerability, co-medication with antibiotics without discontinuation of the study drug, and study medication use of <65% or >150% of the prescribed amount. Up to 200% were acceptable if it could be credibly reassured that a part of the medication had been spilled or used on another child. The mean treatment compliance was more than 100% in both treatment groups. There was no difference between the subgroups of 1- to 3-year old and 4- to 5-year old children.

The participants' demographic and anthropometric characteristics are shown in Table 1. In both groups, slightly more than half of the children were boys. The average age was 3 years in both groups. 359 children (syrup: 248; solution: 111) were between 1 and 3 years and 232 (syrup: 155; solution: 77) were 4 to 5 years old. At baseline, all children suffered from coughing, 395 (syrup, 98.0%) and 186 (solution, 98.9%) exhibited pulmonary rales at auscultation, and 201 (syrup, 49.9%) and 82 (solution, 43.6%) had dyspnea.

**Table 1:** Sample characteristics (safety analysis set/full analysis set; absolute and relative number of patients with chi-square-test *p*-value or mean ± SD with *t*-test *p*-value).

	EPs 7630 syrup (*n* = 403)	EPs 7630 solution (*n* = 188)	*p*-value
Sex			
Male Female	214 (53.1%) 189 (46.9%)	102 (54.3%) 86 (45.7%)	0.79
Age (years)	3.0 ± 1.3	3.0 ± 1.4	0.55
Height (cm)	99.1 ± 11.7	99.0 ± 12.3	0.97
Weight (kg)	15.8 ± 3.9	15.9 ± 3.9	0.82

Previous infections and respiratory diseases during the last 3 months prior to inclusion were reported for about 50% and 15% of the children, respectively. During this period, about 12% of the participants had suffered from gastrointestinal disorders and skin disorders, respectively.

During study participation, 208 children in the syrup group (51.6%) and 91 in the solution group (48.4%) received concomitant medication, and 150 (37.2%) and 75 (39.9%) patients, respectively, took concomitant drugs acting on the respiratory system, notably topical nasal preparations (82 patients, 20.4%; 37 patients, 19.7%), expectorants (*n* = 56, 13.9%; *n* = 31, 16.5%), and cough suppressants (*n* = 43, 10.7%; *n* = 19, 10.1%). Substances used by at least 3% of the children in both groups were xylometazoline (109 patients, 18.4%), acetylcysteine (*n* = 49, 8.3%), noscapine (*n* = 24, 4.1%), salbutamol (*n* = 22, 3.7%), and ambroxol (*n* = 21, 3.6%).

### Adverse events

During the intake of EPs 7630 and 7 days thereafter, AEs were observed in 57 out of 403 patients randomized to syrup (14.1%; 66 events) and in 31 out of 188 patients randomized to solution (16.5%; 37 events). The most frequently reported AEs belong to the MedDRA System Organ Class infections (syrup: 7.2%; solution: 7.4%), gastrointestinal disorders (syrup: 2.7%; solution: 3.2%), or investigations (syrup: 1.0%; solution: 2.7%) (Table 2). Except for investigations, the percentage of patients affected by specific types of events did not differ by more than 1% between EPs 7630 syrup and solution. Reported AEs were otitis media (13 patients, 2.2%), tonsillitis (8 patients, 1.4%), vomiting (9 patients, 1.5%), diarrhea (5 patients, 0.9%) and elevated hepatic enzymes (7 patients, 1.2%). One serious AE, which was assessed to be unrelated to the investigational product, was documented in the solution group when a one-year-old boy was hospitalized with a cerebral commotion due to traumatic head injury. Four AEs led to premature withdrawal from the trial but were judged to be unrelated to EPs 7630 intake.

**Table 2:** Patients with adverse events, any causal relationship, by MedDRA System Organ Classes (safety analysis set, absolute and relative number of patients).

	EPs 7630 syrup (*n* = 403)	EPs 7630 solution (*n* = 188)
Ear and labyrinth disorders	1 (0.2%)	2 (1.1%)
Eye disorders	2 (0.5%)	1 (0.5%)
Gastrointestinal disorders	11 (2.7%)	6 (3.2%)
General disorders and administration site conditions	3 (0.7%)	2 (1.1%)
Infections and infestations	29 (7.2%)	14 (7.4%)
Injury, poisoning, and procedural complications	3 (0.7%)	2 (1.1%)
Investigations	4 (1.0%)	5 (2.7%)
Metabolism and nutrition disorders	1 (0.2%)	–
Renal and urinary disorders	2 (0.5%)	–
Reproductive system and breast disorders	1 (0.2%)	–
Respiratory, thoracic, and mediastinal disorders	3 (0.7%)	–
Skin and subcutaneous tissue disorders	2 (0.5%)	1 (0.5%)
Vascular disorders	1 (0.2%)	1 (0.5%)
Any events	57 (14.1%)	31 (16.5%)

For 12 AEs occurring in 11 patients (1.9% of 591 exposed), the causality assessment was other than “unrelated” (Table 3). Gastrointestinal complaints affected 4 patients (0.7%; 5 events). For these, the causal relationship with EPs 7630 treatment was assessed as unlikely, except for diarrhea in one patient, which was assessed as possible. In 7 patients (1.2%; 7 events), elevations of hepatic enzymes were documented as AEs. In all 7 events, this elevation was probably attributable to the underlying AB caused by viral infection and, thus, a causal relationship with EPs 7630 was assessed as unlikely.

**Table 3:** Patients with suspected cases of adverse drug reactions by MedDRA Preferred Terms (safety analysis set, absolute and relative number of patients). In all cases, causality was assessed as “unlikely”, except for 1 case of diarrhea (“possible”).

	EPs 7630 syrup (*n* = 403)	EPs 7630 solution (*n* = 188)
Hepatic enzyme increased	2 (0.5%)	1 (0.5%)
Aspartate aminotransferase increased	1 (0.3%)	2 (1.1%)
Transaminase increased	–	1 (0.5%)
Diarrhea	1 (0.3%)	2 (1.1%)
Vomiting	–	1 (0.5%)
Upper abdominal pain	–	1 (0.5%)
Any potentially related events	4 (1.0%)	7 (3.7%)

### Vital signs and laboratory measures

Analysis of vital signs showed only minor decreases in the means of heart rate, respiration rate, and body temperature between baseline and treatment end (data not shown).

In 65 children, a clinically relevant increase in CRP as an indicator of the underlying infectious condition was present at baseline but not at treatment end. In 15 children, clinically relevantly increased CRP values were observed at both time points, and in 9 patients at treatment end but not at baseline. One case of CRP elevation on Day 7 was assessed as AE since the investigator did not state an alternative cause. The mean CRP values decreased from 7.3 ± 12.3 mg/dl (mean ± SD) to 3.9 ± 10.5 mg/dl in the syrup group (mean change −3.5 points; 95%CI: −4.9 points, −2.0 points) and from 8.8 ± 15.0 mg/dl to 4.2 ± 10.6 mg/dl in the solution group (mean change −4.5 points; 95%CI: −7.1 points, −1.9 points).

At treatment end, an elevation of at least one hepatic enzyme (ALT, AST, *γ*GT) activity was observed in 4.1% (95%CI: 2.6%, 6.0%) of study participants compared to 5.7% (95%CI: 3.9%, 7.9%) at baseline. Four patients had relevantly elevated liver enzyme values at baseline but not at treatment end, and one at both assessments with only minor changes. There was no upward shift of mean activity values after 7 days of treatment.

### BSS-ped and further symptoms related to respiratory infection

**Figure 2:**
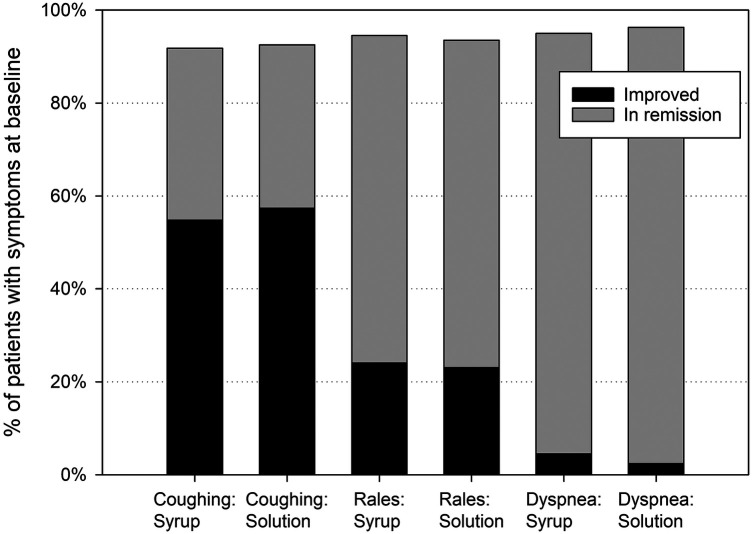
Bronchitis-related symptoms – relative number of patients improved or in remission at treatment end, based on patients with symptoms at baseline.

Following a baseline mean value of 5.7 ± 2.1 points, the BSS-ped total score decreased by 4.2 ± 2.2 points between baseline and treatment end for EPs 7630 syrup and solution combined (full analysis set). Related to the individual symptoms of the BSS-ped (coughing, pulmonary rales at auscultation, and dyspnea), more than 90% of the children were improved or in remission after one week's treatment (Figure 2).

For further symptoms related to respiratory infection (nasal discharge, nasal congestion, sneezing, congestion of pharyngeal mucosa, hoarseness, loss of appetite, sleep disorder), a more or less pronounced decrease from baseline to Day 7 was documented, which was similarly distributed in both groups (Table 4).

**Table 4:** Change of further symptoms related to respiratory infection [Day 7 – baseline, FAS, mean points ± standard deviation (95% confidence intervals); last observation carried forward].

Symptom	EPs 7630 syrup (*n* = 403)	EPs 7630 solution (*n* = 188)	Total
Nasal discharge	−1.09 ± 1.04 (−1.20, −0.99)	−0.94 ± 1.09 (−1.09, −0.78)	−1.04 ± 1.06 (−1.13, −0.96)
Nasal congestion	−0,89 ± 1.04 (−0.99, −0.79)	−0.85 ± 1.03 (−1.00, −0.70)	−0.88 ± 1.04 (−0.96, −0.69)
Sneezing	−0.60 ± 0.86 (−0.69, −0.52)	−0.56 ± 0.80 (−0.68, −0.45)	−0.59 ± 0.84 (−0.66, −0.52)
Congestion of pharyngeal mucosa	−0.77 ± 0.94 (−0.86, −0.68)	−0.76 ± 0.93 (−0.89, −0.62)	−0.76 ± 0.93 (−0.84, −0.69)
Hoarseness	−0.37 ± 0.78 (−0.45, −0.29)	−0.32 ± 0.68 (−0.42, −0.23)	−0.36 ± 0.75 (−0.42, −0.29)
Loss of appetite	−0.70 ± 0.96 (−0.80, −0.61)	−0.66 ± 0.96 (−0.80, −0.53)	−0.69 ± 0.96 (−0.77, −0.61)
Sleep disorder	−0.84 ± 1.01 (−0.94, −0.74)	−0.76 ± 1.02 (−0.90, −0.61)	−0.81 ± 1.01 (−0.90, −0.73)

### General health status and satisfaction with treatment

At treatment end, the investigators reported complete recovery for 273 of the 591 (46.2%) children and major improvement for 223 (37.7%) children according to the IMOS (Table 5). The IMOS ratings performed by the parents indicated complete recovery for 229 (38.7%) and major improvement for 243 (41.1%) children. According to the IMPSS, the parents of 509 (86.1%) patients expressed that they were satisfied or very satisfied with the treatment.

**Table 5:** Therapy outcome using the Integrative Medicine Outcomes Scale (IMOS) at Day 7 (full analysis set, absolute and relative number of patients).

Parameter	Outcome	EPs 7630 syrup (*n* = 403)	EPs 7630 solution (*n* = 188)
Therapy outcome (assessed by the investigator)	Complete recovery	188 (46.7%)	85 (45.2%)
	Major improvement	152 (37.7%)	71 (37.8%)
	Slight to moderate improvement	44 (10.9%)	22 (11.7%)
	No change	9 (2.2%)	6 (3.2%)
	Deterioration	7 (1.7%)	3 (1.6%)
	Missing	3 (0.7%)	1 (0.5%)
Therapy outcome (assessed by the parents)	Complete recovery	159 (39.5%)	70 (37.2%)
	Major improvement	160 (39.7%)	83 (44.1%)
	Slight to moderate improvement	52 (12.9%)	22 (11.7%)
	No change	22 (5.5%)	8 (4.3%)
	Deterioration	7 (1.7%)	5 (2.7%)
	Missing	3 (0.7%)	0 (0.0%)

The results for the full analysis set were fully confirmed in the per-protocol analysis set (data not shown). Differences in symptom improvement between both treatment groups were negligible.

## Discussion

The taste of medications is essential for children's acceptance of orally administered drugs (29, 30). This is a major reason why syrup formulations play an important role in the pediatric population. For EPs 7630, which was already demonstrated to be efficacious in children from 1 year of age and in adults suffering from ARTIs (13–18, 21, 23, 31–33), the syrup offers a sugar-free, non-alcoholic option which is especially suitable for pediatric patients. The present post-authorization study in compliance with an obligation of the German Drug Regulatory Authority investigated the safety and tolerability of the EPs 7630 syrup in comparison to the EPs 7630 solution, which has been marketed for many years, in 1- to 5-year-old children suffering from AB. As viral ARTIs in children may cause an elevation of hepatic enzymes according to literature reports (3, 34–37), a monitoring of hepatic enzyme activities was carried out. Expectedly, the results of our study indicate that the syrup has an equally beneficial safety profile as the long-established solution; no indication of more severe or previously unknown types of reactions was seen.

Suspected cases of adverse drug reactions were observed in less than 2% of the study participants. These events were gastrointestinal disturbances and elevated hepatic enzymes, and, in all cases, causality was assessed as “unlikely”, except for 1 case of diarrhea (“possible”). Noteworthy, the percentage of patients with elevated hepatic enzyme activities was higher at baseline than after the treatment interval. All elevations were below the limit of 5-fold of upper limit of normal and therefore below the threshold which is considered as indicative of a liver injury (38). All cases of increased enzyme activities were most probably associated with the underlying or a concomitant viral infection.

The effects of viral infections on hepatic enzymes (AST, ALT, *γ*GT) were recently investigated in 1,010 children and adolescents aged 1 to 17 years suffering from ARTIs (3). An elevation of at least one hepatic enzyme was observed in 11.1% (95%CI: 9.2%, 13.3%) of subjects at the time of study entry. At follow-up after 3 to 7 days, hepatic enzymes elevation was found in 11.2% (95%CI: 9.2%, 13.4%) of the participating patients. In 7.1% (95%CI: 5.5%, 9.0%) of patients, an elevation of at least one hepatic enzyme value was present at both study entry and follow-up.

It can therefore be concluded that the liver enzyme activity elevations reported in the present study reflect the incidence in a population of children with ARTIs. This interpretation is consistent with the investigators' assessment of AEs. It is further supported by the fact that the percentage of patients with elevated values at the end of the trial was lower than that observed at baseline with no evidence of any upward shift in transaminase mean values.

Mild nasal or gingival bleeding and hypersensitivity reactions, which are labelled as rare adverse effects in the patient information leaflet, were each observed once in this study. In both cases, a causal relationship with the investigational treatment was excluded. Noteworthy, about half of the children in both groups used concomitant drugs. Some of them may also cause adverse effects like those observed during the present study, e.g., gastrointestinal complaints or hypersensitivity reactions. Overall, our study results do not suggest any safety signal, nor do they indicate any previously unknown risks associated with EPs 7630 in pre-school children.

The primary goal of the study was to evaluate the safety and tolerability of a 7-day treatment with EPs 7630 syrup in comparison to the solution. For this open label safety study, the data were analyzed in an exploratory way. The open label design and lack of a placebo control are a limitation of the present investigation. This was chosen, because the trial was not intended for demonstrating treatment efficacy and effects under treatment. Our results must therefore be interpreted within the scope of this setting. Nevertheless, the extent of symptom relief measured with the BSS-ped, IMOS and IMPSS was similar to that observed in double-blind, randomized, controlled clinical trials in children suffering from acute bronchitis, in which EPs 7630 was shown to be significantly superior to placebo (23).

During the one-week treatment with EPs 7630 syrup or solution, more than 90% of the participating children exhibited a clinically meaningful alleviation or even complete remission of AB symptoms. This effect was evident to the treating physicians as well as to the infants' parents and it was also reflected by a significant decrease of serum CRP as an objective inflammatory marker. The recovery rate was in the range of those observed during other placebo-controlled trials (31, 33). EPs 7630 syrup and solution were equally safe and well tolerated in pre-school children suffering from AB, for which the therapeutic efficacy of the herbal extract has already been demonstrated.

## Data Availability

The datasets presented in this article are not readily available because raw data cannot be shared both due to ethical reasons and to data protection laws. To the extent permitted by law, the data required for validation purposes have already been disclosed in results reports on corresponding data bases. All relevant data are within the paper. Requests to access the datasets should be directed to Prof. Wolfgang Kamin, paediatrie.hamm@valeo-kliniken.de.

## Footnotes

^1^EPs^®^ 7630 is the active substance of Umckaloabo^®^ by Dr. Willmar Schwabe GmbH & Co. KG, Germany.
